# Predicting worse survival for newly diagnosed T cell lymphoma based on the decreased baseline CD16−/CD16 + monocyte ratio

**DOI:** 10.1038/s41598-020-64579-z

**Published:** 2020-05-08

**Authors:** Wei Zhang, Jing Ruan, Daobin Zhou, Xiao Han, Yan Zhang, Wei Wang, Mingqi Ouyang

**Affiliations:** Department of Hematology, Peking Union Medical College Hospital, Beijing, 100730 China

**Keywords:** Prognostic markers, T-cell lymphoma

## Abstract

T cell non-Hodgkin lymphoma (T-NHL) is highly invasive and heterogeneous without accurate prognosis prediction. We proposed peripheral CD16−/CD16 + monocytes the additional indicators for T-NHL prognosis. We prospectively recruited 31 T-NHL patients without previous treatment. The CD16−/CD16 + monocyte ratio before chemotherapy was calculated and regular follow up was performed to calculate prognostic prediction value. Tumor associated macrophages (TAM) in tumor tissue were counted and transcriptome sequencing of CD16− and CD16 + monocytes was applied to explore potential mechanisms. We found that T-NHL patients had higher ratio of total monocytes especially the CD16 + monocytes along with a decreased ratio of CD16−/CD16 + monocytes, compared to the health control. The 1-year overall survival rate was 0.492 and 0.755 for CD16− monocyte/CD16 + monocyte ratio of <11 and ≥11(p < 0.05), respectively. The peripheral CD16−/CD16 + monocyte ratio was significantly relevant with the pathological CD68/CD206 macrophage ratio. The differently expressed genes in CD16− and CD16 + monocytes from T-NHL patients were mainly involved in signaling molecules related to tumor microenvironment. Pro-tumor genes were identified in monocyte subsets especially in CD16 + monocytes. In conclusion, the ratio of peripheral CD16−/CD16 + monocyte helps to stratify the prognosis of T-NHL. The relatively increased CD16 + monocytes may contribute to the pro-tumor microenvironment of T-NHL.

## Introduction

T cell lymphoma is one kind of non-Hodgkin’s lymphoma with high incidence in Asia^1^. There are no effective treatment strategies despite its high invasiveness, heterogeneity and mortality. Accurate prediction and evaluation of the prognosis of T-cell non-Hodgkin lymphoma (T-NHL) helps to select the correct treatment strategies to improve the curative effect and survival^2^. However, still a high-risk subset of patients could not be recognized based on previous clinical prognostic index including PIT (Prognostic Index of Peripheral T cell Lymphoma). Over the past decade, researchers have been seeking to identify novel biomarkers including immunohistochemistry-based detection, gene expression profiling and liquid biopsy^3^.

Tumor microenvironment especially the tumor-associated macrophage (TAM) has been recognized as a promising predictive marker in lymphoma^4^ and peripheral blood monocytes are considered the major sources of TAMs. In a recent study we found for the first time that the lower CD16− monocytes/CD16− monocytes ratio seemed to indicate the worse prognosis of diffuse large B cell lymphoma (DLBCL)^5^. Therefore, we prostitute CD16−/CD16 + monocytes may reflect tumor microenvironment and may also be used to predict the survival of T-NHL.

In this prospective cohort study, we recruited T-NHL patients to identify the value of peripheral CD16−/CD16 + monocyte ratio in predicting the survival of T-NHL. The correlation between peripheral monocyte subsets and TAMs in the pathological tissue and the different gene expression profiles of monocyte subsets between the patients and the health were also explored in order to reveal the potential mechanisms that monocyte subsets played in the prognosis of T-NHL.

## Results

### CD 16-/CD16 + monocytes predict the prognosis of T cell lymphoma

31 T cell lymphoma patients without treatment were enrolled in this study. The clinical characteristics were presented in Table 1. Standard treatment regimens were given including CHOP (cyclophosphamide, doxorubicin, vincristine and prednisone) and SMILE (dexamethasone, methotrexate, ifosfamide, L-asparaginase and etoposide). Until June 2018, the median follow-up was 14 months (1–18months) while the 1-year OS (overall survival) was 0.645 and the 1-year PFS (progression-free survival) was 0.516 for the whole cohort. At the end of the follow-up, 11 patients reached CR (complete remission), 3 reached PR (partial remission), 2 was SD (stable disease), 3 had PD (progressive disease) and 12 died all from disease progression.

**Table 1:** The clinical characteristics of 31 T-NHL patients enrolled in the prospective study.

Clinical characteristics		number	percentage
Age	<60	25	80.6%
	≥60	6	19.4%
Subtype	NK/T nasal type	11	35.5%
	PTCL, NOS	5	16.1%
	ALCL, ALK-	4	12.9%
	ALCL, ALK +	3	9.7%
	SPTL	4	12.9%
	Others^a^	4	12.9%
Ann Anbor stage	I~II	6	19.4%
	III~IV	25	80.6%
Extranodal number	0, 1	15	48.4%
	>1	16	51.6%
ECOG	0, 1	18	58.1%
	>1	13	41.9%
LDH	normal	13	41.9%
	elevated	18	58.1%
BM involvement	No	24	77.4%
	Yes	7	22.6%
EBV infection	No	17	54.8%
	Yes	14	45.2%

^a^Others: 1 AITL, 1 EATL, 1 ANKL and 1 Sezary.

^b^Abbreviations: PTCL, NOS: peripheral T-cell lymphoma, not otherwise specified; ALCL: anaplastic large cell lymphoma; ALK: anaplastic lymphoma kinase; SPTL: subcutaneous panniculitis-like T-cell lymphoma; AITL: angioimmunoblastic T-cell lymphoma; EATL: epitheliotropic intestinal T-cell lymphoma; ANKL: aggressive natural killer-cell leukemia; BM: bone marrow;

**Figure 1:**
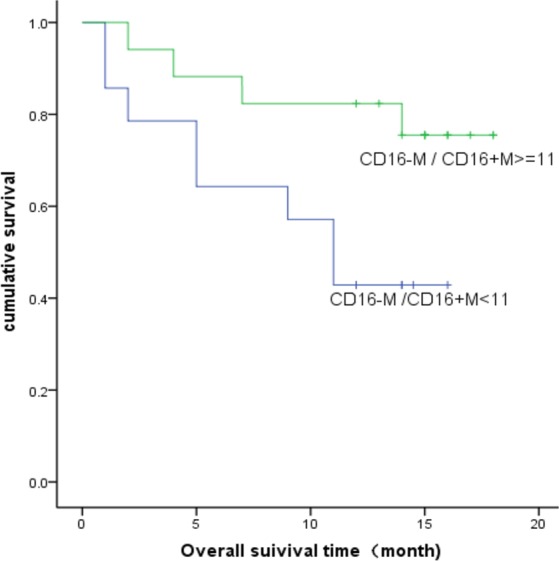
Kaplan-Meier survival curves for overall survival. The 1-year overall survival rate was 0.492 and 0.755 for CD16− monocyte/CD16 + monocyte ratio of <11 and ≥11 respectively.

**Figure 2:**
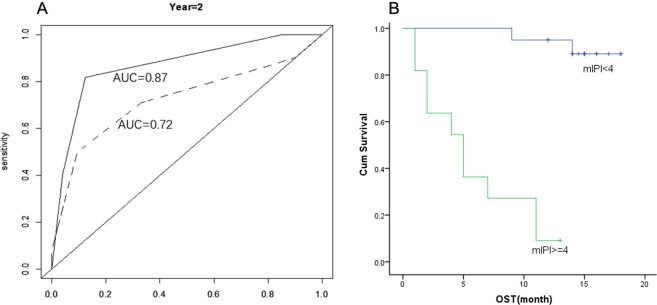
The Survival analysis using a model constructed by CD16−/CD16 + monocytes and IPI (mIPI score system). (**A**) The survival receiver operating characteristic (ROC) curve of 2-year-survival for CD16−/CD16 + monocytes ratio and IPI model. AUC was 0.87, which was significantly higher than IPI (dash line) alone. (**B**) The estimated 2-year-survival was 89.1 ± 7.3% and 9.1 ± 8.7% for mIPI <4 and ≥4 respectively.

The numbers of monocyte subsets and the differences between the patients and the health were displayed in Table 2. Compared to healthy controls, the T-NHL patients had significantly higher percentage and absolute number of CD16 + monocyte and a decreased ratio of CD16−/CD16 + monocytes. No differences were found between patients with and without EBV infections nor bone marrow involvement. We performed single factor analysis (see Supplementary Tables S1 and S2) for OS and PFS for known prognostic variables including IPI (International Prognostic Index) and monocyte subsets. ECOG (Eastern Cooperative Oncology Group performance status) number, bone marrow involvement, IPI and the absolute number of CD16− monocyte were related to the OS while Ann Abor Stage, EOCG, LDH (lactate dehydrogenase) and IPI were predictors for PFS. Kaplan–Meier analysis of overall survival showed that the 1-year overall survival rate was 0.492 and 0.755 for CD16− monocyte/CD16 + monocyte ratio of <11 and ≥11 (p < 0.05), respectively (Fig. 1). Considering the sample size and the event number of our study, the CD16−/CD16 + monocyte ratio was adjusted by IPI using Cox analysis. We found that this ratio could independently predict the prognosis of T-NHL (Table 3). Furthermore, mIPI score system was then constructed combining CD16−/CD16 + monocyte ratio and IPI (age ≥ 60 y, Ann Abor stage III-IV, ≥ 2 extra nodal involvement sites, ECOG ≥ 2, elevated serum LDH). In order to determine the prognostic value of this ratio, the time-dependent receiver operating characteristic (ROC) curve of 2-year-survival was performed. The AUC was 0.87. The specificity was 87.7% and the sensitivity was 81.7% when the cut-off value was 4. The Kaplan-Meier analysis of overall survival was then performed with the cut-off value determined by survivalROC. We found that the estimated 2-year-survival was 89.1 ± 7.3% and 9.1 ± 8.7% for mIPI <4 and ≥4 respectively (Fig. 2).

**Table 2:** The number of monocyte subsets in T-NHL patients and the health control.

	control	T-NHL	P value
Sex (male:female)	17:14	17:14	
Age	42.5	43.9	
Monocyte (X10^9^)	0.43 ± 0.10	0.52 ± 0.31	0.125
Monocyte (%)	6.58 ± 0.98	8.28 ± 4.21	0.036
CD16− monocyte (X10^9^)	0.41 ± 0.09	0.48 ± 0.29	0.229
CD16− monocyte (%)	6.30 ± 0.96	7.53 ± 3.86	0.096
CD16 + monocyte (X10^9^)	0.018 ± 0.006	0.044 ± 0.033	<0.001
CD16 + monocyte (%)	0.28 ± 0.07	0.75 ± 0.5	<0.001
CD16− monocyte/D16 + monocyte	24.44 ± 8.12	12.97 ± 8.11	<0.001

**Table 3:** Prognostic models for OS and PFS constructed with IPI and CD16−/CD16 + monocyte ratio.

	β	95% CI	p
**Multi-COX for OS**			
IPI	3.821	1.822–8.012	0.000
CD16−M/CD16 + M	0.255	0.074–0.880	0.031
**Multi-COX for PFS**			
IPI	2.380	1.430–3.961	0.001
CD16−M/CD16 + M	0.301	0.106–0.856	0.024

### The peripheral monocytes subsets correlate with the tumor associated macrophages

**Figure 3:**
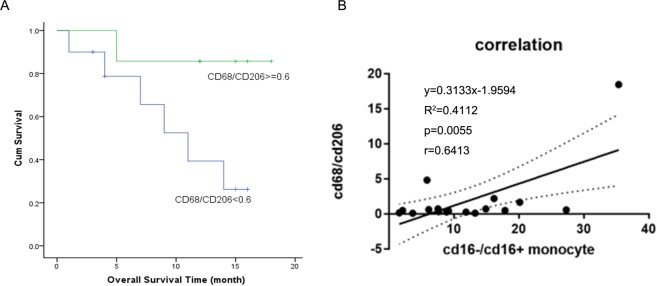
Role of CD68/CD206 macrophages in predicting T-NHL overall survival and its relationship with CD16−/CD16 + monocytes. (**A**) Kaplan–Meier analysis of overall survival indicated that the 1-year overall survival rate was 0.857 ± 0.132 for patients with tissue CD68/CD206 macrophages ratio ≥0.6, which was significantly better than those with low CD68/CD206 macrophages ratio (0.263 ± 0.158). (**B**) Pearson analysis indicated a correlation between tissue CD68/CD206 macrophages and peripheral CD16−/CD16 + monocytes (p = 0.006, r = 0.6413).

Multiplexed immunofluorescent staining including CD68 and CD206 and multi-spectral imaging were performed for the tumor tissues from 17 T-NHL patients without chemotherapy. The basic clinical characteristics were shown in Supplementary Table S3. 88.2% of them were Ann Anbor III-IV. 16.32 ± 14.49% of the cells were CD68 positive while 29.05 ± 21.04% of the cells expressed CD206. The CD68/CD206 ratio was calculated for each patient and the Kaplan–Meier analysis of overall survival indicated that patients with tissue CD68/CD206 macrophages ratio ≥0.6 had better prognosis (Fig. 3A). The 1-year overall survival were 0.263 ± 0.158 and 0.857 ± 0.132 independently. We then explored the relationship between peripheral monocyte and tissue macrophages. Interestingly, Patients with low peripheral CD16−/CD16 + monocytes usually had low tumor CD68/CD206 macrophages and had worse prognosis. Pearson analysis were performed, and we found the ratio of peripheral CD16−/CD16 + monocytes was correlated with tissue CD68/CD206 macrophages (p = 0.006, r = 0.6413) (Fig. 3B).

### Transcriptional profiles of the peripheral monocytes for T-NHL patients

**Figure 4:**
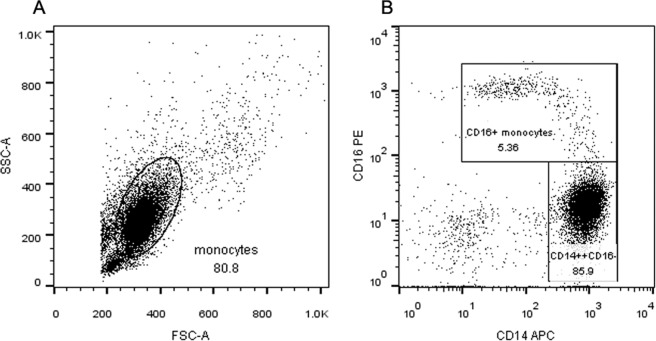
The gating strategy for monocyte subsets sorting. (**A**) The flow cytometry of total cells after depletion of non-monocytes from PBMC by pan monocyte isolation kit. The total monocytes were gated. (**B**) The CD14 ++ CD16− and CD16 + monocytes were gated from total monocytes according to CD14 and CD16 expression level.

**Figure 5:**
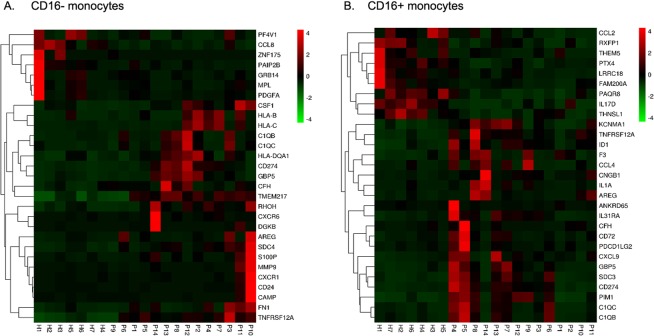
The heatmap of genes involved in signal transduction expressed by CD16− and CD16 + monocytes. The transcriptional profiles were apparently differently between the health (H1 to H7) and the T-NHL patients (P1 to P14) both in CD16− (**A**) and CD16 + monocytes (**B**).

The total peripheral monocytes of 7 health controls and 14 T-NHL patients were isolated and were sorted into CD16− and CD16 + subsets (Fig. 4) for transcriptome sequencing. Log2 fold change over 2 with Q value below 0.001 counted by DEGseq were defined as significantly differentially expressed genes. 1481 and 1937 differentially expressed genes were identified in CD16− and CD16 + monocytes respectively between the patients and the health. KEGG pathway analysis revealed that signaling molecules involved in cancer development and immune system were major categories. Heatmap was performed and genes involved in signal transduction were displayed (Fig. 5). In brief, both CD16− and CD16 + monocytes of T-NHL patients had increased expression of *C1QC*, *C1QB*, *CD274(PDL1)*, *GBP5* and *TNFRSF12A*. *HLA-B*, *HLA-C*, *HLA-DQA1* were highly expressed in CD16− monocytes in patients while *SDC3*, *KCNMA1*, *IL31RA*, *PDCD1LG2*(*PDL2*), *CXCL9*, *ID1*, *AREG*, *CD72*, *PIM1*, especially *CCL4* were highly expressed in CD16 + monocytes compared to the control.

Genes differentially expressed between CD16− and CD16 + monocytes were also compared between the patients and the controls. *B3GNT7*, *TIAM2*, *NT5DC4*, *XPNPEP2*, *TMPRSS3*, *ACY3* were upregulated only in T-NHL patients in CD16 + monocytes.

## Discussion

3 subsets of monocytes have been identified including the classical monocytes (CD14 + + CD16−), the intermediate monocytes (CD14 + + CD16+) and the nonclassical monocytes (CD14 + CD16 ++), the latter two were defined as CD16 + monocytes^6^. Previous studies indicated circulating CD16 + monocytes predicted prognosis in solid tumors^7^. Our team also found for the first time that the lower CD16− monocytes/CD16 + monocytes ratio was related to worse survival in DLBCL without known mechanisms^5^. In this study, we explored the clinical value of monocyte subsets in T-NHL and the potential mechanisms. We found that compared to the health control, the T-NHL patients had higher total monocytes especially the CD16 + monocytes. We also verified the lower CD16−/CD16 + monocytes ratio corresponded to poorer prognosis in T-NHL. Moreover, mIPI score system combining the clinical characteristics and biological behaviors was built to better predict the prognosis of T-NHL.

Monocyte in the peripheral blood could be recruited to the tumor tissue by several chemo-attractants and polarize to M1 or M2 type of macrophage. It is known that the M1 macrophage is mainly related to inflammation and phagocytosis while the M2 type is mainly involved in immunosuppressive process, fibrous formation and tissue repair. M2 macrophages express CD163, CD206 and in a narrow sense are TAMs^8^. Whether CD16− and CD16 + monocytes play different roles in TAM differentiation and anti-tumor effects remains controversial. CD16− monocytes mainly involves in inflammatory phagocytosis reactions and are considered the major source of tumor associated macrophages^9^. The function of CD16 + monocytes *in vivo* remains unknown. *In vitro* models indicated angiogenesis for these cells and a higher propensity to become dendritic cells^10^. Clinical studies revealed a significantly elevation of CD16 + monocytes in malignant disease^11^. Tumor promotion characteristics of CD16 + monocytes were identified in cholangiocarcinoma patients with higher expression of adhesion molecules (CD11c, CD163) and angiogenic factor-related genes (VEGF-A, epiregulin)^7^. Since immunosuppressive and pro-tumorigenic TAMs (M2 macrophage) also express higher CD163, CD206 and VEGF, we wondered the relationship between peripheral monocyte subsets and TAMs in the pathological tissue. Multiplexed immunofluorescent staining was performed and lower peripheral CD16−/CD16 + monocytes ratio was found to in correspondence with lower M1/M2 macrophages by Pearson analysis. The intrinsic mechanisms that relatively increased CD16 + monocytes paralleled more M2 macrophages remains unknown. One postulation is that CD16 + monocytes are prone to be induced to TAMs by tumor cells^11–13^. Cytokines produced by CD16 + monocytes may also promote the conversion of TAM^14,15^.

To further understand the characteristics of monocyte subsets in T-NHL patients, the transcription profiles were sequenced and compared. Interestingly, we found that CD16− and CD16 + monocytes in T-NHL patients both seemed to be tumor-promoting while the latter to be more robust. They both had increased expression of C1q encoding genes (*C1QC*, *C1QB*), *TNFRSF12A* and IFN-γ induced genes (*PD-L1*, *GBP5*). C1q was previously thought to be the activator of classical complement pathway^16^ and was recently found to be cancer-promoting independently in the tumor microenvironment^17^. Functional convergence was revealed between C1q and TNFα in phagocytosis, chemotaxis, apoptosis and angiogenesis^18^. Tumor necrosis factor receptor superfamily member (*TNFRSF12A*) may serve a role in tumor growth and metastasis and has been reported to be elevated in several types of cancer^19^. PD-L1 expression in tumor microenvironment was important in immune evasion and was considered a promising treatment target. *In vitro* model showed PD-L1/PD-L2 expressing monocytes/TAMs suppressed activation of PD-1^hi^ NK cells in Hodgkin lymphoma^20^ and *in vivo* study also found the PD-L1(+) monocytes effectively suppressed tumor-specific T cell immunity^21^. It is inspiring that we found the significantly higher expression of PD-L1 in T-NHL monocytes and PD-L2 (*PDCD1LG2*) in CD16 + monocytes since it may help predict the PD-1/PD-L1 blockage treatment response in T-NHL. Pro-tumor genes including *SDC3*^22,23^, *KCNMA1*^24^, *IL31RA*^25^, *ID1*^26,27^, *AREG*^28^, *PIM1*^29^ and *CCL4*^30^ were also highly expressed in CD16 + monocytes in T-NHL patients compared to the health which was not found in CD16− monocytes. Notably, high expression of CCL4 was verified to induce the filtration of TAM in colon cancer in a recent study^31^ and it is reasonable to postulate that increased CD16 + monocytes in T-NHL help to recruit TAMs by increased secreted CCL4. Genes differentially expressed between CD16− and CD16 + monocytes may also play important roles in tumor progression especially those absent in health controls. *B3GNT7*, *TIAM2*, *NT5DC4*, *XPNPEP2*, *TMPRSS3*, *ACY3* were found to be significantly highly expressed in CD16 + monocytes only in patients. *B3GNT7*, *TIAM2* and *TMPRSS3* were all found to promote the proliferation, invasion and migration of cancer cells. *XPNPEP2* is a proline hydrolytic enzyme that hydrolyzes peptides to cause a loss of substrate activity and finally helps tumor metastasis in the microenvironment^32^. To sum up, pro-tumor genes involved in microenvironment were identified in both CD16− and CD16 + monocytes, and some chemokines secreted by CD16 + monocytes may help induce TAM infiltration which may explain why the decreased ratio of CD16−/CD16 + monocytes indicated poorer prognosis and may be the promising therapeutic targets.

There are some limitations in our study. The sample size should be enlarged considering the heterogeneity of T-NHL and a longer time follow up should be done in the future. Besides, further direct *in vitro* and *in vivo* studies should be conducted to verify the function of differentially expressed genes in tumor microenvironment and to help explore the role of monocyte subsets involved in the recruiting and polarization of TAMs.

## Conclusions

The ratio of peripheral CD16−/CD16 + monocytes help to stratify the prognosis of T-NHL. The relatively increased CD16 + monocytes may contribute to the pro-tumor microenvironment of T-NHL.

## Methods

### Patient and controls

T-NHL Patients over 18 years old were enrolled during September 2015 to May 2017 who were first diagnosed to be T-NHL in Peking Union Medical College Hospital (PUMCH) and had not been treated before. All cases were classified according to the 2008 World Health Organization (WHO) classification of hematopoietic malignancies and the pathology was confirmed by two independent pathologists. Matched healthy volunteers were recruited from the physical examination center of PUMCH at the same time. All patients signed informed consent and the study was approved by the Ethics Committee of Peking Union Medical College Hospital (No: ZS1163). All procedures were done in accordance with the tenets of the Declaration of Helsinki.

### Clinical data, specimen collection and follow-up

The following parameters were collected for T-NHL patients: age, sex, subtype, Eastern Cooperative Oncology Group (ECOG) performance status (PS), Ann Arbor stage (I-IV), presence of B symptoms, number and type of involved sites, LDH, EBV-DNA copies, prognostic index including International Prognostic Index (IPI), according to their medical record in PUMCH. The peripheral blood and/or formalin fixed paraffin-embedded (FFPE) tissues were obtained before treatment. All patients were treated and followed up regularly in PUMCH. During the follow up, treatment response was evaluated by enhanced computed tomography or PET-CT (positron emission tomography-computed tomography). Complete remission (CR), partial remission (PR), stable disease (SD), progressive disease (PD) and relapse were defined according to previously published reports.

### Flow cytometry analysis

Blood samples were collected for leukocyte subsets differential using the Cytodiff™ flow cytometric system developed by Beckman Coulter. This system includes flow cytometry (FC500), premixed Cytodiff reagent and CytoDiff CXP analysis software. Six antibodies including CD36-FITC, CD2-PE, CD294-PE, CD19-ECD, CD16−PC5, and CD45-PC7 were mixed in the Cytodiff reagent. Analysis was performed according to the manufacturer’s instructions. In brief, 10 μL of Cytodiff reagent was mixed with 100 μL whole blood. After 20 min incubation at room temperature, lysing solution (Versalyse solution; Beckman Coulter) was added to lyse the red blood cells for 15 min. Fluorescent microspheres (Flow-Count Fluorospheres, Beckman Coulter) were used to perform count of each cell type. Approximately 20,000 cells were acquired and analyzed automatically by the analysis software. The analysis software is self-gating and separates populations by automatic logic pathways^33^.

### Multiplexed immunofluorescent staining

FFPE sections 3 um thick were stained by de-paraffinisation, epitope retrieval, endogenous peroxidase blockade and secondary antibody detection. Tyramide-signal amplification (TSA) based system (PerkinElmer, Waltham, Massachusetts, US) was used for fluorescent staining allowed for repeated staining steps. Rabbit antibodies against CD68(1:20000) with opal570 and CD206(1:1500) with opal650 were used. Each slide was then treated with DAPI and then washed in PBST. Slides were air dried, mounted with anti-fade fluorescence mounting medium (#0100-01, southernbiotech) and stored in dark at 4 °C prior to imaging. The Vectra automated multispectral imaging system (PerkinElmer, Massachusetts, USA) was used to perform both low (x4) and high (x20) power scans of 10 randomly selected tissue grids. The individual biopsy TIL density per region of interest (ROI) was determined from 10 randomly selected ROIs of tumor or stromal areas for each section.

### RNA quantification of the monocyte subsets

Peripheral blood mononuclear cells (PBMC) were extracted from the peripheral blood by Ficoll-Paque PLUS (GE healthcare, US). Pan Monocyte Isolation Kit (Miltenyi Biotec, Germany) was used to remove non-monocytes, including T cells, NK cells, B cells, dendritic cells, and basophils, which were indirectly magnetically labeled using a cocktail of biotin-conjugated antibodies and Anti-Biotin MicroBeads. The highly pure unlabeled monocytes were obtained by depletion of the magnetically labeled cells. Within the pan monocyte population, CD 14 + +CD16− and CD16 + monocytes were sorted according to their expression level of CD14 and CD16 by Aria III after incubated with anti-CD14 and anti-CD16 antibodies (BioLegend, US). 0.5 ml Trizol was added to the cells and incubated at room temperature for 3 min. Total RNA was extracted with chloroform (0.1 ml) and precipitated with isopropanol (0.4 ml) before washed with 75% ethanol. The RNA pellet was finally dissolved in 10ul RNase-free water. RNA concentration, RIN, 28 S/18 S,and size were detected by Agilent 2100 Bioanalyzer (Agilent RNA 6000 Nano Kit) and the purity was tested by NanoDrop^TM^. Total RNA sample was amplified by Clontech SMARTer™ PCR cDNA Synthesis Kit. The library was quantitated and amplified on cBot to generate the cluster on the flowcell. And the amplified flowcell was sequenced pair end on the HiSeq. 2000 System, read length 50. Mapping of reads to the human genome (hg19) was then performed.

### Statistical analysis

Most statistical analysis was carried out using SPSS 19.0 software and the statistical programming language R (www. r-project.org). Qualitative variables were analyzed by Pearson’s test or by the Kruskal-Wallis test. Spearman’s test was used to test correlations between different variables. All P-values are based on two-tailed statistical analysis, considering P-values below 0.05 as significant. Specificity, sensitivity and cut-off were established using receiver operating characteristic (ROC) curve analysis. Statistical analysis and ROC curves were performed with the aid of the survivalROC R package^34^. Overall survival (OS) and progression-free survival (PFS) were estimated using the Kaplan–Meier method and two-tailed log-rank test. Multivariate analysis was performed using the Cox’s proportional-hazards model. As for RNA quantification, normalization and test for differential expression was performed in R with the DESeq package. Expression levels are given in FPKM (fragments per kilobase of transcript per mllion fragments mapped). FPKM value of 0.05 was set as the lower bound in our analyses. KEGG pathway information was obtained from https://www.genome.jp/ and phyper R package was applied for enrichment analysis. Heatmap was performed using the OmicShare tools, a free online platform for data analysis (http://www.omicshare.com/tools).

## Supplementary information

Supplementary Information.

## Data Availability

The datasets generated during and/or analyzed during the current study are available from the corresponding author on reasonable request.
