# Supplementary material for: Predicting worse survival for newly diagnosed T cell lymphoma based on the decreased baseline CD16−/CD16 + monocyte ratio
Source: Sci Rep. 2020 May 8;10:7757. doi: 10.1038/s41598-020-64579-z (PMC7211003; doi:10.1038/s41598-020-64579-z)
Supplement: Supplementary file 1 — Supplementary Information. [file 41598_2020_64579_MOESM1_ESM.pdf]

Supplementary Information

Predicting worse survival for newly diagnosed T cell lymphoma based on the decreased baseline CD16-/CD16+ monocyte ratio

Wei Zhang[1¶], Jing Ruan[1¶], Daobin Zhou[1*], Xiao Han[1], Yan Zhang[1], Wei Wang[1], Mingqi Ouyang[1]

Supplementary Table S1: Hazard ratios of prognostic factors for overall survival in T-NHL

	Overall survival (OS)		
**Risk factor**	**HR**	**95% CI**	**p**
**Age（≥60y vs <60y）**	1.944	0.523-7.222	0.321
**Sex（male vs female）**	1.817	0.547-6.038	0.330
**Stage**	4.3	0.799-23.16	0.089
**B symptoms (yes vs no)**	2.625	0.334-20.62	0.359
**Number of extra nodal sites (>1 vs 0,1)**	0.913	0.294-2.835	0.875
**ECOG (2,3,4 vs 0,1)**	*7.303*	*1.943-27.45*	*0.003*
**BM involved (yes vs no)**	*3.614*	*1.135-11.50*	*0.030*
**LDH elevated (yes vs no)**	4.540	0.989-20.850	0.052
**IPI**	*3.58*	*1.767-7.257*	*0.000*
**CD16-monocyte(X10^9)(>0.3 vs <0.3)**	*0.252*	*0.075-0.843*	*0.025*
**CD16-monocytes/CD16+monocytes(>11 vs <11)**	0.320	0.096-1.072	0.065
**neutrophil/monocytes**	*1.023*	*1.006-1.041*	*0.008*
**neutrophil/monocytes(>12 vs <12)**	*3.757*	*1.183-11.937*	*0.025*
**neutrophil/CD16- monocyte**	*1.015*	*1.004-1.027*	*0.009*
**neutrophil/CD16- monocytes(>12 vs <12)**	*3.757*	*1.183-11.937*	*0.025*

Supplementary Table S2: Hazard ratios of prognostic factors for progressive-free survival in T-NHL

	Progression-free survival (PFS)		
**Risk factor**	**HR**	**95% CI**	**p**
**Age（≥60y vs <60y）**	0.996	0.282-3/527	0.997
**Sex（male vs female）**	2.563	0.898-7.312	0.079
**Stage**	*3.632*	*1.148-11.49*	*0.028*
**B symptoms (yes vs no)**	1.271	0.349-4.636	0.716
**Number of extra nodal sites (>1 vs 0,1)**	0.762	0.293-1.986	0.579

**ECOG (2,3,4 vs 0,1)**	*2.989*	*1.098-8.141*	*0.032*
**BM involved (yes vs no)**	2.001	0.699-5.724	0.196
**LDH elevated (yes vs no)**	*4.966*	*1.415-17.429*	*0.012*
**IPI**	*2.169*	*1.316-3.575*	*0.002*
**CD16-monocyte(X10^{9})(>0.3 vs <0.3)**	0.442	0.149-1.308	0.140
**CD16-monocytes/CD16+monocytes(>11 vs <11)**	0.432	0.162-1.150	0.093
**neutrophil/monocytes**	*1.015*	*1.002-1.028*	*0.028*
**neutrophil/monocytes(>12 vs <12)**	2.221	0.766-6.437	0.143
**neutrophil/CD16- monocyte**	*1.010*	*1.001-1.019*	*0.027*
**neutrophil/CD16- monocytes(>12 vs <12)**	2.221	0.766-6.437	0.142

Supplementary Table S3: The basic clinical characteristics of T-NHL patients for multiplexed immunofluorescent staining

Clinical characteritics		number	percentage
**Age**	<60y	13	76.5%
	≥60y	4	23.5%
**Sex**	Male	10	58.8%
	Female	7	41.2%
**subtype**	NK/T lymphoma, nasal	7	41.2%
	PTCL, NOS	2	11.8%
	ALCL， ALK-	2	11.8%
	ALCL， ALK+	2	11.8%
	SPTL	2	11.8%
	AITL	2	11.8%
**Ann Anbor**	I~II	2	11.8%
	III~IV	15	88.2%
**LDH**	Normal	5	29.4%
	Elevated	12	70.6%
**ECOG**	0,1	11	64.7%
	2,3,4	6	35.3%
**BM involvement**	Yes	3	17.6%
	no	14	82.4%
**Tissue source**	Lymphoid nodal	3	17.6%
	Nasal tissue	5	29.4%
	Cutaneous tissue	5	29.4%
	Bone marrow	2	11.8%
	others	2	11.8%
